# Aging enhances pro-atrogenic gene expression and skeletal muscle loss following respiratory syncytial virus infection

**DOI:** 10.1007/s11357-024-01370-2

**Published:** 2024-10-02

**Authors:** J. Sophie Sagawe, Verity I. P. Loake, Peter J. M. Openshaw, Paul Kemp, Fiona J. Culley

**Affiliations:** National Heart and Lung Institute, Faculty of Medicine, Imperial College London, St Mary’s Campus, Norfolk Place, London, W2 1PG UK

**Keywords:** Respiratory, Infection, Aging, Muscle

## Abstract

Aging and many age-related health conditions are associated with skeletal muscle loss. Furthermore, older adults are more susceptible to severe respiratory infections, which can in turn lead to muscle wasting. The mechanisms by which respiratory viral infection can impact skeletal muscle in older adults are not well understood. We determined the effects of acute infection with respiratory syncytial virus (RSV) on the lung and skeletal muscle of aged mice. RSV infection caused more severe disease in aged mice with enhanced weight loss, reduced feeding, higher viral load, and greater airway inflammation. Aged but not young mice showed decreased leg muscle weight at the peak of illness and decreased size of leg muscle fibers. Aged mice increased muscle-specific expression of atrophy-promoting enzymes (Atrogin-1 and MuRF-1) and failed to increase the rate of muscle protein synthesis during RSV infection. In aged mice, the changes in Atrogin-1 and MuRF-1 gene expression in skeletal muscle correlated with IL-6 levels in the lungs. These findings indicate that RSV infection of aged mice provides a model for studying the diverse adverse systemic consequences of respiratory viral infections on health and wellbeing in older adults.

**Supplementary information:**

The online version contains supplementary material available at 10.1007/s11357-024-01370-2.

## Introduction

The population of the world is aging rapidly and infection with respiratory viruses is a major cause of morbidity and mortality in older adults [1]. Although often associated with severe bronchiolitis in infants, RSV infection causes a substantial and often underestimated disease burden in those over 65 years of age [2–5]. Risk factors for severe lower respiratory tract RSV disease in adults include advanced age and chronic age-related conditions, including frailty and respiratory and circulatory diseases [6].

In addition to local inflammation in the lung, there is a growing awareness that respiratory viral infections can elicit systemic effects on multiple organs, including skeletal muscle [7]. Myalgia is a symptom of influenza and RSV infection in the elderly population [8, 9] and Covid-19 has been associated with both acute and post-acute changes to skeletal muscle, including muscle weakness, pain, and fatigue [10, 11]. Elevated serum creatine kinase, which is released following muscle damage, is associated with worse outcomes in adults admitted to the intensive care unit with influenza infection and in Covid-19 [12–15]. Furthermore, muscle inflammation, which corresponded with the duration of illness, was found in post-mortem studies of patients with Covid-19 [15]. The mechanisms by which respiratory viral infection can lead to changes in muscle mass and function are not fully understood and may result from a combination of the inflammatory response, hypoxia, and changes in physical activity, muscle innervation and nutrition [10, 16].

Changes to skeletal muscle leading to loss of muscle mass and strength occur with advancing age (sarcopenia) [1, 17] and in age-related respiratory conditions, such as chronic obstructive pulmonary disease (COPD) [18–20]. This may in part relate to the baseline elevated inflammation (“inflamm-aging”) and inappropriate and elevated inflammatory responses in older adults, and pro-inflammatory cytokines, such as IL-6, may promote muscle loss [21]. Furthermore, acute sarcopenia may be precipitated in older adults by relatively minor acute stresses that are associated with increased inflammation and muscle disuse, including illness and surgery [22–24]. Skeletal muscle protein is continuously turned over, existing in a constant balance between synthesis and catabolism. A small imbalance of these processes results in hypertrophy or atrophy [25]. Increased expression of genes associated with muscle protein breakdown, and decreased expression of muscle growth-promoting genes has been reported in aged mice infected with Influenza A virus [26]. However, the interaction between aging, acute respiratory viral infection and muscle loss is not fully understood.

Older mice develop a more severe respiratory infection in response to RSV than younger mice [27–30]. In this study, we used this observation to develop a model of muscle wasting in response to respiratory infection in aged mice. We measured the lung inflammatory response, muscle fiber size, protein synthesis and muscle gene expression over the course of RSV infection in aged mice and compared to the same parameters in young mice following infection in order to evaluate the effect of RSV infection on muscle loss in aged animals.

## Methods

### RSV propagation and infection of young and aged mice

RSV strain A2 (ATCC) was propagated in human epithelial type 2 (HEp-2) cells and viral titre determined using an immunoplaque assay, as described [31]. Young (10–12-week-old) and aged (80–85-week-old) female C57BL/6 mice were purchased from Charles River Laboratories (Margate, United Kingdom), housed in individually ventilated cages, and provided with food and water *ad libitum*. All experiments were performed under Home Office license in accordance with the United Kingdom’s Home Office standards under the Animals (Scientific Procedures) Act, 1986 and local Animal Welfare and Ethical Review Board approval. Mice were infected intranasally with 2.3 × 10^5^ plaque forming units of RSV in 75 μl under isofluorane anesthesia. Age-matched control groups were not infected. Mice were weighed daily and monitored for signs of illness. Food hoppers and water bottles were weighed daily to determine average food and water intake per mouse.

### Tissue collection

Mice were euthanized either when uninfected or at 8- or 18-days post-infection (dpi). The trachea was cannulated, and the lungs flushed three times with 1 mL of 5 mM EDTA PBS to obtain bronchoalveolar lavage (BAL) fluid. BAL fluid was centrifuged, and the supernatant stored at -80 °C. Total protein was measured in the BAL fluid using Bradford reagent, with reference to a standard curve of bovine serum albumin (BSA), according to manufacturer’s instructions (Sigma-Aldrich, St Louis, MO, USA). The left lung was extracted and used for flow cytometric analysis. The tibialis anterior (TA) muscle was extracted from both legs and weighed.

### Puromycin incorporation assay

Protein synthesis was determined according to puromycin incorporation in an adaptation of published protocols [32, 33]. 30 min prior to euthanasia, mice were injected intraperitoneally with 0.8 μmol puromycin dihydrochloride (Sigma-Aldrich) in 100 μl PBS. After euthanasia, the TA muscle was extracted and homogenized in a TissueLyser LT (Qiagen, Manchester, UK) in cOmplete protease inhibitor buffer (Roche, Welwyn Garden City, UK) at 50 Hz for 4 min. After centrifugation, the supernatant protein content was determined using Bradford reagent, as described above. 96 well plates were coated overnight with supernatant diluted to 100 ng protein / μl in bicarbonate buffer (pH 9.6). The next day plates were blocked with 5% BSA in PBS for 30 min, then incubated with 100 ng/ml of puromycin antibody (clone 12D10, Merck Millipore) for one hour at room temperature. Plates were washed with PBS and incubated with the secondary antibody, AffiniPure peroxidase conjugated goat anti-mouse IgG2a (1:1000, Jackson Laboratories), for one hour at room temperature. 3,3′,5,5′-Tetramethylbenzidine (TMB) substrate (Life Technologies) was added, and the reaction stopped with 1N HCl. Absorbance was read at 450 nm on an FluoStar Omega plate reader (BMG Lifetech).

### ELISA

IL-6 protein in BAL fluid was measured using an R&D Systems DuoSet kit (DY406-05) according to the manufacturer’s instructions.

### Immunohistochemistry

Left TA muscles were embedded in OCT compound (VWR, Dublin, Ireland) and snap frozen in isopentane cooled with liquid nitrogen. Cryosections of 6 µm thickness were cut using a cryostat microtome (Bright Instruments, Luton, UK). Cryosections were thawed, permeabilized with 0.5% Triton X-100 (Sigma-Aldrich Ltd, Gillingham, UK) for five minutes, and blocked in 2% goat serum for an hour. Slides were incubated with primary antibodies (anti-myosin heavy chain I (BA-D5; 1:50), anti-myosin heavy chain IIA (SC-71; 1:100), anti-myosin heavy chain IIB (BF-F3; 1:50), and anti-laminin (L9393; 1:100; Sigma-Aldrich)) overnight and secondary antibodies (goat anti-mouse IgG2b-Alexa Fluor 555 (1:200; Invitrogen, Loughborough, UK), goat anti-mouse IgG1-Alexa Fluor 350 (1:200; Invitrogen), goat anti-mouse IgM-Alexa Fluor 488 (1:200; Invitrogen), F(ab’)2-goat anti-rabbit IgG (H + L)-Alexa Fluor 647 (1:200; Invitrogen)) for one hour. Primary antibodies against myosin heavy chain epitopes deposited by Schiaffino, S.were obtained from the Developmental Studies Hybridoma Bank created by the NICHD of the NIH and maintained at the University of Iowa, Department of Biology, Iowa City, IA 52242. Slides were mounted in ProLong Gold mountant (Life Technologies, Loughborough, UK) and imaged on a Zeiss Axio Observer inverted widefield microscope. An entire section taken from the middle of the muscle was imaged at 20X magnification using a motorized stage with a 5 μm overlap between images. Images were analysed using Fiji software using a macro script [34]. In brief, the macro carried out the following steps: The channel containing only the laminin staining was isolated and converted to a binary image, distinguishing extracellular matrix from individual muscle fibers. This separated muscle fibers sufficiently to allow for automatic object (fiber) detection and batch measurement. The minimum Feret’s diameter was measured for each fiber in the image. The mean minimum Feret’s diameter of all muscle fibers was calculated for each mouse.

### Flow cytometry for cell counts

To obtain lung cells, lung tissue was homogenized using a gentleMACS dissociator (Miltenyi Biotec Ltd, Woking, UK) and digested in 2.5 mL collagenase XI (Sigma-Aldrich) at 37 °C for 30 min. BAL cells were obtained following centrifugation of the BAL fluid. For both lung and BAL cell suspensions, red blood cells were lysed in filter-sterilized ACK buffer (154 mM ammonium chloride, 10 mM potassium bicarbonate, 0.1 mM EDTA), and single-cell suspensions obtained by filtering through 100 µm filters. Final cell suspensions were in RPMI 1640 (Thermo Fisher Scientific, Waltham, MA, USA) with 10% fetal calf serum (Gibco, Waltham, MA), 100 U/ml penicillin and 100 μg/ml streptomycin (Sigma-Aldrich, St Louis, MO, USA). Live cell numbers were determined by flow cytometry following 7-AAD viability staining (Biolegend, UK) using CountBright Absolute Counting Beads (Thermo Fisher Scientific). Flow cytometry was carried out on an LSRFortessa™ Cell Analyzer (BD Biosciences). Data was acquired using BD FACSDiva™ software (BD Biosciences) and analyzed with FlowJo software version 10.1 (TreeStar). For differential cell counts, BAL cells were spun onto glass slides using a Cytospin 4 cytocentrifuge and stained using Kwik-Diff staining according to manufacturer’s instructions (Thermofisher). Cells were identified as neutrophils, eosinophils, large mononuclear cells (macrophages) or smaller mononuclear cells (monocytes/lymphocytes). At least 300 cells were counted per slide using an Eclipse TS100 Light microscope (Nikon).

### Quantitative real-time PCR

Lung tissue and TA muscle were homogenized in TRIzol (Thermo Fisher Scientific) with a TissueLyser LT (QIAGEN), and RNA extracted according to manufacturer’s instructions. RNA concentration was measured using a NanoDrop® ND-1000 spectrophotometer (Thermo Fisher Scientific). cDNA was synthesized using a high-capacity RNA-to-cDNA kit (Applied Biosystems) according to the manufacturer’s instructions. mRNA expression was assessed by real-time quantitative PCR (qPCR) using TaqMan Universal MasterMix II with no UNG (Applied Biosystems), run on a 7500 Fast Real-Time PCR System machine (Applied Biosystems). Gene expression was normalized to GAPDH expression and the fold difference in expression compared to young uninfected mice was determined as 2^−ΔΔCT^, except for the RSV L-gene, which was quantified using a DNA plasmid standard curve to obtain copy number [35]. For the IL-6 PCR analysis, where expression of IL-6 was not detected in some samples, these samples are shown using a Ct value of 40 cycles to calculate the maximum possible relative expression. Primer – probe sets were purchased from Thermo Fisher: GAPDH (*Gapdh*, Mm99999915_g1), Atrogin-1 (*Fbxo32*, Mm00499523_m1), MuRF-1 (*Trim63*, Mm01185221_m1), Myostatin (*Mstn*, Mm01254559_m1), Myogenin (*Myog*, Mm00446194_m1), IGF-1 (*Igf1*, Mm00439560_m1), Pax7 (*Pax7*, Mm01354484_m1), IL-6 (*Il6*, Mm00446190_m1).

### Statistical analysis

Data are shown as means with the standard error of the mean. Statistical significance of differences between groups was determined with unpaired, two-tailed Student’s t-tests, or one- or two-way ANOVA with Bonferroni’s post-test, where appropriate, to correct for multiple comparisons. For PCR relative expression, data were normalized by log2 transformation before statistical analysis. Data were analyzed with GraphPad Prism 6.0 software. Values of p ≤ 0.05 were considered statistically significant. *p ≤ 0.05, **p ≤ 0.01, ****p ≤ 0.001, ****p ≤ 0.0001.

## Results

### Aged mice have higher viral loads and display increased lung and airway inflammation following RSV infection

**Fig. 1:**
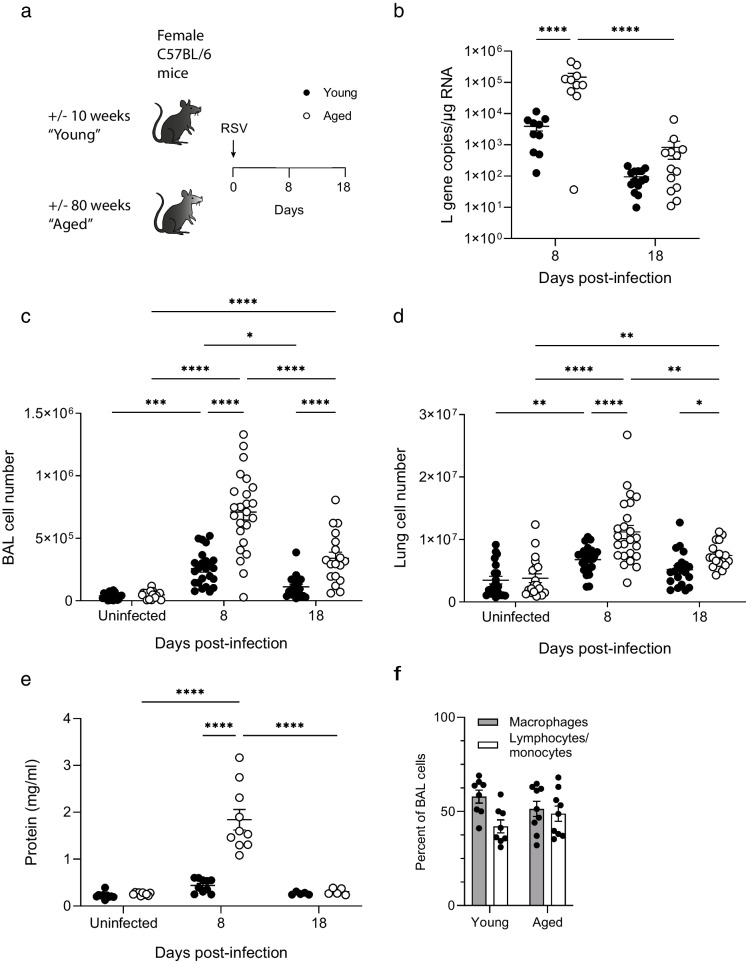
Aged mice develop more severe RSV disease. (**a**) Young and aged female C57BL/6 mice were infected with RSV. Tissues were harvested 8- or 18-days post-infection (dpi) or from age-matched uninfected controls. (**b**) RSV L gene copy number quantified by qPCR in lung tissue. 8 dpi *n* = 10, 18 dpi *n* = 13–14. (**c**) Live cells in bronchoalveolar lavage (BAL) fluid and (**d**) lung tissue of young and aged mice infected with RSV. Uninfected controls *n* = 20; 8 dpi *n* = 24; 18dpi *n* = 18 and 20. (**e**) Total protein content in BAL fluid. Uninfected controls *n* = 10; 8 dpi *n* = 10; 18 dpi *n* = 5. (**f**) Differential cell counts of BAL cells. Data are presented as individual mice and mean ± SEM with young as filled circles and aged as open circles. The statistical significance of differences was determined using 2-way ANOVA with Bonferroni’s multiple comparisons test

To characterize how age affects RSV disease in our hands at the dose of RSV used, young and aged female C57BL/6 mice were infected intranasally (Fig. 1a). Mice were culled 8 or 18 days after infection (acute and recovery phases). Uninfected young and aged mice provided baseline measures. We quantified RSV L gene copy number in lung tissue by qPCR. No L gene was detectable in the lungs of either young or aged uninfected mice (not shown). At day 8, significantly more copies of L gene were detectable in the lungs of aged mice compared to young mice (Fig. 1b).

Flow cytometry was used to count cells in homogenized lung tissue and in the airways using bronchoalveolar lavage (BAL) fluid. In both young and aged mice, the number of cells in BAL fluid and lung tissue increased significantly from baseline to the peak of illness at day 8 (Fig. 1c, d). At day 8, aged mice had significantly more cells in their BAL fluid and lung tissue than young mice. At day 18, cell numbers had reduced but were still higher in aged than in young mice. Cell numbers in the BAL fluid and lung tissue of aged mice remained significantly higher at day 18 compared to baseline, whereas numbers in young mice were not significantly different between uninfected mice and at day 18. This demonstrates that airway inflammation in RSV infection in aged mice is characterized by an exaggerated cellular recruitment to the airways. Total protein in the BAL fluid increased significantly in aged mice with infection and was significantly higher in aged mice than young mice at day 8 returning to baseline by d18. These results add further evidence that aged mice have higher levels of lung inflammation than young mice after RSV infection. Differential counts were performed on cytospin preparations of BAL cells from d8 of RSV infection (Fig. 1f). This demonstrated that the inflammation in the airways of both young and aged mice predominantly consisted of a lymphocyte/monocyte infiltration and the proportion of these inflammatory cells in the BAL fluid was not statistically significantly different between young and aged mice. Due to the greater extent of inflammation in aged mice, the total number of lymphocytes/monocytes was higher in the BAL fluid of aged mice (mean of 1.6 × 10^5^ in young vs 4.7 × 10^5^ in aged mice, *p* = 0.0012, unpaired t-test). These data confirm the greater inflammatory response in aged mice than in young mice to RSV infection.

### Aging leads to enhanced weight loss and greater inappetence during RSV infection

**Fig. 2:**
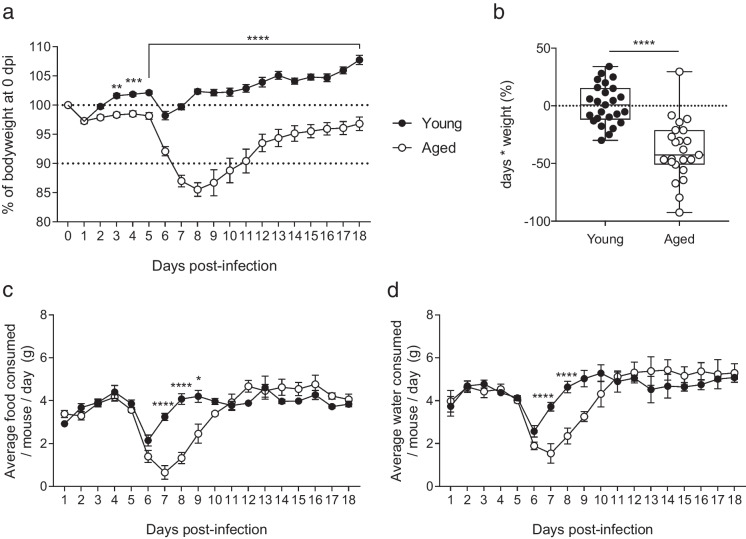
Weight loss, food and water consumption of young and aged mice infected with RSV. Young and aged female C57BL/6 mice were infected with RSV. (**a**) Bodyweight as a percentage of weight on day of infection with RSV (day 0). 0–5 dpi *n* = 55 young and 54 aged mice; 5–8 dpi *n* = 30 young and 29 aged; 8–18 dpi *n* = 20 young and 18 aged mice. (**b**) Area under the curve of weight loss up to day 8 after infection as a percentage of starting weight. *n* = 25 young and 23 aged mice. (**c**) Food and (**d**) water intake per mouse after RSV infection. 0–5 dpi *n* = 45 young and 44 aged mice; 5–8 dpi *n* = 20 young and 19 aged; 8–18 dpi *n* = 15 young and 13 aged mice. Data are presented as group means ± SEM with young as filled circles and aged as open circles. The statistical significance of differences was determined using Student’s two-tailed unpaired t test or 2-way ANOVA with Bonferroni’s multiple comparisons test

Aged mice infected with RSV started losing weight approximately 6 days after infection, peaking at day 8 (Fig. 2a). By day 18, the aged mice had still not returned to their baseline weight. At this dose young mice did not lose weight, although with higher doses young mice can lose weight following RSV infection [36]. Weight as a percentage of day 0 bodyweight was significantly different between young and aged mice from day 3 to day 18 of infection. To quantify the relative weight loss of young and aged mice, the area under the curve (AUC) of percentage bodyweight over 8 days of RSV infection was calculated. Aged mice had a significantly lower (more negative) AUC compared to young mice (Fig. 2b).

Acute illness is often associated with reduced food intake. We measured food and water intake over the course of infection by weighing food hoppers and water bottles daily (Fig. 2c, d). Food intake was reduced in both groups following infection corresponding to the peak in weight loss and was significantly lower in aged mice compared to young mice on days 7, 8 and 9. Water intake also reduced in both groups and was significantly lower in aged mice than young on days 7 and 8.

### Aging leads to muscle wasting after RSV infection

**Fig. 3:**
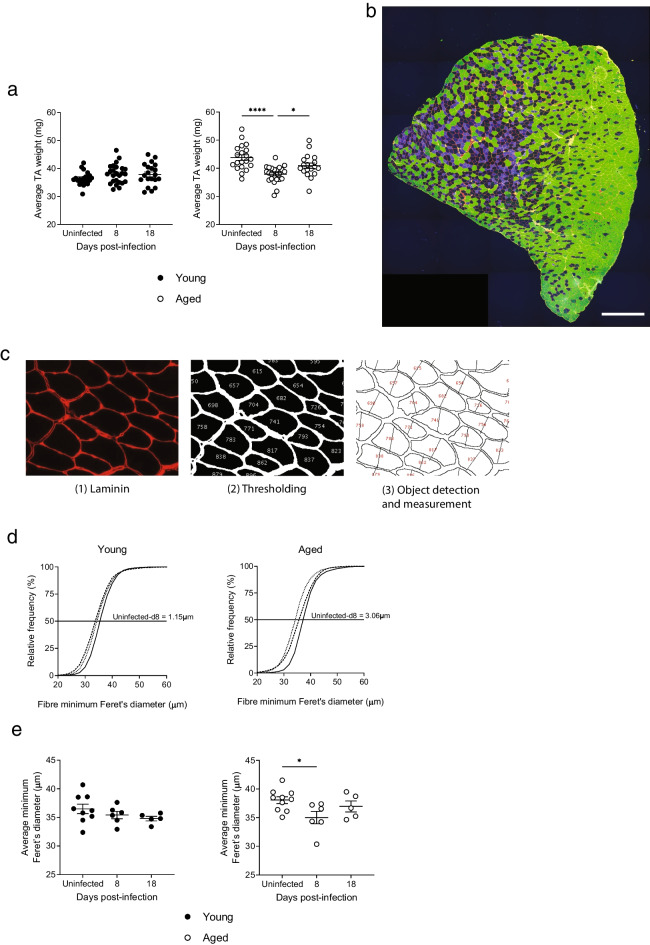
Aged mice lose skeletal muscle mass during RSV infection. Young and aged female C57BL/6 mice were infected with RSV. (**a**) Tibialis anterior muscle weight (average of both TA muscles per mouse). Data are presented as individual mice and mean ± SEM. Uninfected *n* = 20; 8 dpi *n* = 25; 18 dpi *n* = 18 and 20. (**b**) Tiled image of a cross section of tibialis anterior muscle, scale bar = 500 μm. Colors indicate staining with antibodies to myosin heavy chain I (yellow), myosin heavy chain IIA (blue), myosin heavy chain IIB (green) and laminin (red). (**c**) Steps of the image analysis (1) Isolation of the laminin stain channel (2) Thresholding of the laminin image creating a binary mask that distinguishes muscle fibers from extracellular matrix (3) Detection of the individual muscle fibers and measurement of minimum Feret’s diameter. The white scale bar represents 50 μm. (**d**) Cumulative frequency histograms of the TA muscle fiber minimum Feret’s diameter measured in *n* = 10 young and *n* = 9 aged mice either uninfected (continuous line), 8 dpi (dotted line), or 18 dpi (dashed line). The difference between the minimum Feret’s diameter at 50% relative frequency of uninfected mice and 8 dpi are shown. (**e**) Average minimum Feret’s diameter of TA muscle fibers. Data are presented as individual mice mean ± SEM with young as filled circles and aged as open circles. Uninfected *n* = 9 and 10; 8 dpi *n* = 6; 18 dpi *n* = 5. The statistical significance of differences in (**a**) and (**e**) was determined using one-way ANOVA with Bonferroni’s multiple comparisons test

The weight lost by aged mice during RSV infection is likely to include muscle mass, fat mass, and water weight. To interrogate the effect of infection on the loss of muscle mass specifically, the tibialis anterior (TA) muscle was extracted from both legs of young and aged mice infected with RSV and weighed. Prior to infection, aged mice had heavier TA muscles than young mice, consistent with their higher baseline bodyweight (Fig. 3a). At the peak of infection at day 8, the TA weight of aged mice had decreased significantly from baseline. 18 days after infection, the average TA weight of aged mice remained significantly lower than in uninfected aged mice. In contrast, the TA weight of young mice remained almost unchanged throughout infection.

### Aged mice display smaller muscle fiber sizes after RSV infection

To measure the size of muscle fibers directly, the TA muscle of young and aged mice infected with RSV were cryosectioned and the sections stained by immunohistochemistry (Fig. 3b). Sections were stained for myosin heavy chain I, IIA and IIB, and laminin, a part of the basal lamina in the extracellular matrix surrounding the bundles of muscle fibers. Representative images of TA muscles from all groups are shown in the Supplemental data. Following imaging, the minimum Feret’s diameter of each muscle fiber in the section was measured based on the laminin staining (Fig. 3c). Minimum Feret’s diameter was chosen as the most appropriate measure of fiber size, since it is less prone to distortion by irregular cutting angles than maximum diameter or cross-sectional area. The cumulative frequency distribution of the fiber minimum Feret’s diameter was calculated for young and aged mice at baseline, and after 8 and 18 days of RSV infection (Fig. 3d). At baseline, aged mice displayed larger muscle fiber sizes than the young. 8 days after RSV infection, the frequency distributions of TA fiber size of aged mice had decreased compared to uninfected aged mice resulting in a shift in the frequency distribution of 3.06 μm at 50% relative frequency, compared to a difference of only 1.15 μm in the young (Fig. 3d). 18 days after RSV infection, aged mice display larger muscle fibers than at d8. The average fiber minimum Feret’s diameter for individual mice decreased significantly at day 8 in aged mice, but not in the young (Fig. 3e). Together, this demonstrates that aged mice exhibit muscle wasting during RSV infection, whereas at the dose of RSV used in this model, young mice do not.

### The muscles of aged mice have a pro-atrogenic gene expression signature following respiratory viral infection

To determine whether there were intrinsic changes in pathways regulating protein synthesis and degradation in the skeletal muscle during infection, gene expression in TA muscle was measured by qPCR and expression was determined following normalization to a housekeeping gene and is shown as expression relative to young, uninfected mice. 2-way ANOVA was used to compare expression between experimental groups, including between time points of the same age group and between young and aged mice at each time point. The genes measured included three genes encoding products that promote muscle atrophy; *Fbxo32* (encoding Atrogin-1), *Trim63* (encoding MuRF-1), and *Mstn* (encoding myostatin) and three genes encoding products that promote muscle growth; *Myog* (encoding myogenin), *Pax7* (encoding Paired box 7 (Pax7)), and *Igf1* (encoding insulin-like growth factor 1 (IGF-1).

**Fig. 4:**
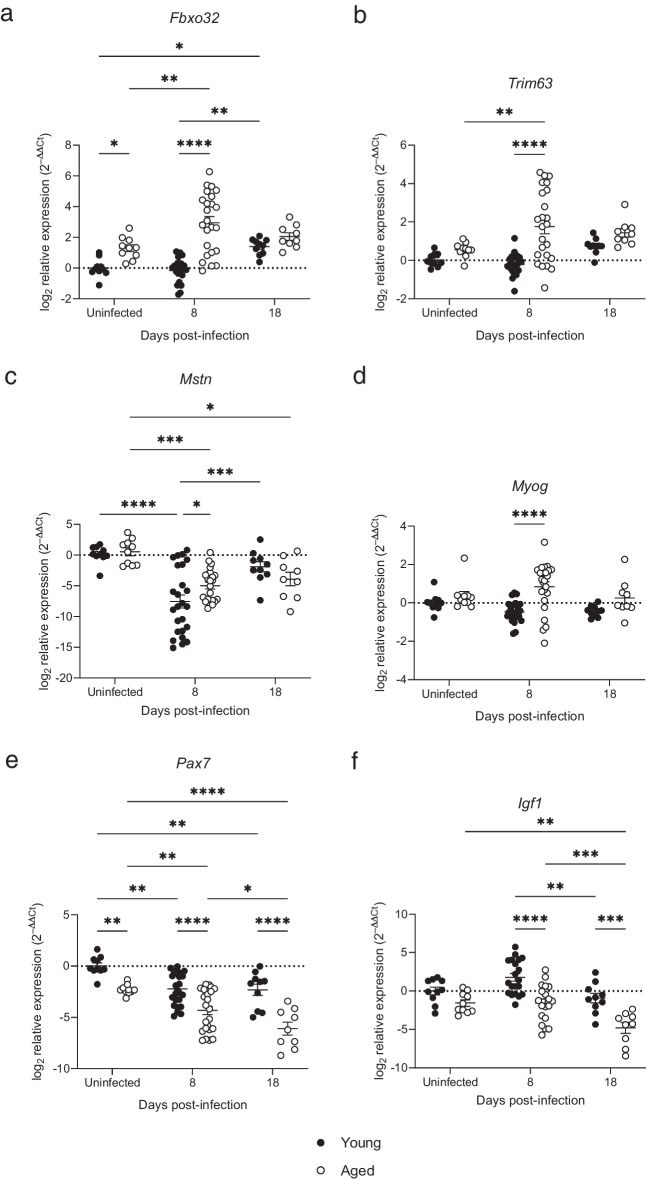
Aged mice display enhanced expression of atrophy-promoting genes during RSV infection. Gene expression in TA muscle was assayed by RT-qPCR, normalized to *Gapdh* expression, and shown as the log2 of expression relative to young uninfected mice (2^−ΔΔCt^). (**a**) *Fbxo32* encoding Atrogin-1. (**b**) *Trim63* encoding MuRF-1. (**c**) *Mstn* encoding Myostatin. (**d**) *Myog* encoding Myogenin. (**e**) *Pax7* encoding Pax7. (**f**) *Igf1* encoding IGF-1. Data are presented as individual mice and mean ± SEM with young as filled circles and aged as open circles. 0 and 18 dpi *n* = 9 and 10; 8 dpi *n* = 5 and 20. The statistical significance of differences was determined using 2-way ANOVA with Bonferroni’s multiple comparisons test

The muscle specific E3 ubiquitin ligases Atrogin-1 and MuRF-1 ubiquitinate several muscle-specific proteins, including actin, myosin, desmin, and MyoD1, causing their degradation in 26S proteasomes and reduction in muscle mass and fiber size. The expression of *Fbxo32* was higher in uninfected aged mice than young mice at baseline (Fig. 4a), but *Trim63* and *Mstn* were no different (Fig. 4b, c). 8 days after infection, expression of both *Fbxo32* and *Trim63* was significantly higher than at baseline in the muscles of older mice, and this expression was significantly higher than expression in young, infected mice (Fig. 4a, b). In contrast, *Mstn* expression decreased from baseline to day 8 of RSV infection in the muscles of both young and aged mice and was significantly lower in young mice on day 8 post infection. By day 18 post infection, there was no significant difference in expression of *Fbxo32*, *Trim63* or *Mstn* in young and aged mice. However, *Fbxo32* expression in the young was elevated on day 18 post infection compared to baseline, and *Mstn* expression remained significantly lower at day 18 than at baseline in the aged mice (Fig. 4c).

For the genes that promote muscle growth, in uninfected mice, *Pax7* expression was significantly lower in aged mice than in the young (Fig. 4e). Following infection, *Myog* expression was significantly greater in the muscles of aged mice than the muscles of young mice on day 8 (Fig. 4d). *Pax7* expression was significantly lower following infection in both young and aged mice compared to uninfected mice, and expression was significantly lower in the aged mice (Fig. 4e). *Igf1* expression was significantly lower in aged compared to young mice at both 8 and 18 days after infection (Fig. 4f). By day 18 post infection, expression of *Myog* did not differ between young and aged mice and was not significantly different to baseline. In contrast, expression of both *Pax7* and *Igf1* was significantly lower in the aged mice than the young at 18 days post infection. *Pax7* expression remained lower on day 18 than at baseline for the young mice. For both *Pax7* and *Igf1*, in the aged mice expression declined further by day 18 when compared to day 8 post infection (Fig. 4e and f), such that by day 18 *Igf1* expression was significantly lower than the baseline value.

### Aging reduces polypeptide synthesis in skeletal muscle during respiratory infection

**Fig. 5:**
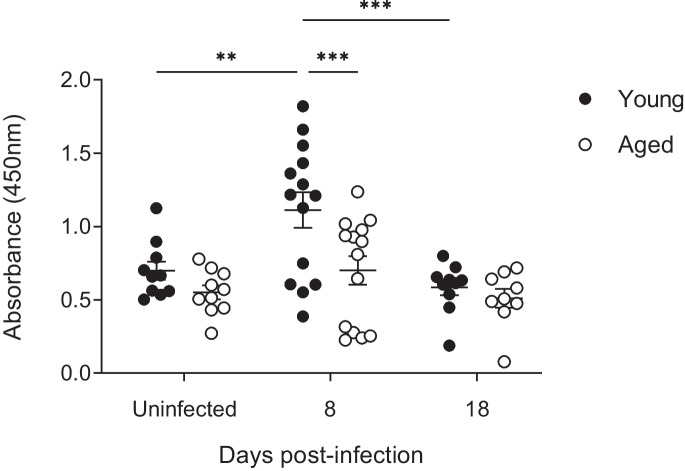
Polypeptide synthesis in skeletal muscle is lower in aged than in young mice following RSV infection. Young and aged female C57BL/6 mice were infected with RSV. Incorporation of puromycin into TA muscle was determined at d8 and d18 dpi. Data are presented as individual mice and mean ± SEM with young as filled circles and aged as open circles. Uninfected *n* = 10; 8 dpi *n* = 14; 18 dpi *n* = 9 and 10. The statistical significance of differences was determined using 2-way ANOVA with Bonferroni’s multiple comparisons test

Muscle is a highly dynamic tissue and muscle mass is the net result of protein catabolism and synthesis. Puromycin incorporation into nascent polypeptide chains can be used as a correlate of the rate of protein synthesis [32, 33, 37]. Mice were injected intraperitoneally with puromycin dihydrochloride 30 min prior to culling and the puromycin content of TA muscles was measured by ELISA (Fig. 5). Surprisingly, levels of puromycin incorporation increased significantly with infection in young mice but not in the aged. This suggests that protein synthesis activity in skeletal muscle increases during respiratory infection in young but not aged mice. At the peak of infection at day 8, puromycin incorporation, and by extension overall protein synthesis, was significantly lower in aged mice than in young mice. By day 18, levels had returned to baseline in both young and aged mice. This discrepancy in upregulation of protein synthesis at the peak of infection may contribute towards the higher loss of muscle mass observed in aged mice.

### Muscle atrogenic signals correlate with lung interleukin-6 (IL-6) in aged mice

**Fig. 6:**
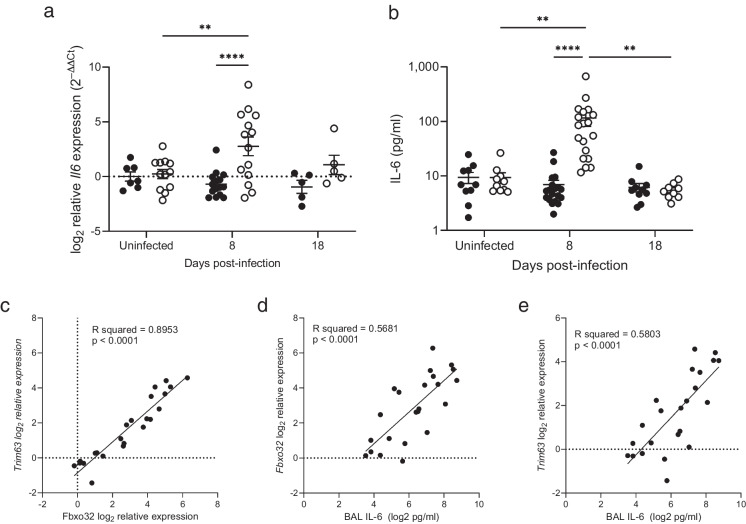
Airway IL-6 correlates with atrogenic gene expression in the muscles of aged mice. (**a**) IL-6 gene expression in the muscle (uninfected controls *n* = 7 and 12, d8 *n* = 15 and 14, d18 *n* = 5) and (**b**) IL-6 protein measured by ELISA in BAL fluid (uninfected controls *n* = 10; 8 dpi *n* = 19; 18 dpi *n* = 9 and 10) in RSV infected young and aged mice. Data are presented as individual mice and mean ± SEM with young as filled circles and aged as open circles. The correlation in aged, RSV infected mice between (**c**) *Fbxo32* and *Trim63* expression in TA muscle; (**d**) BAL IL-6 and expression of *Fbxo32* in TA muscle and (**e**) BAL IL-6 and expression of *Trim63* in TA muscle. The dotted line represents the mean expression in uninfected young mice

Muscle wasting can be associated with inflammation. We measured expression of the pro-inflammatory cytokine IL-6 in the muscle of RSV infected aged and young mice (Fig. 6a). Expression of *Il6* was elevated in aged but not young mice on d8 of infection and returned to baseline levels of expression by d18. The protein levels of the pro-inflammatory cytokine IL-6 were assayed in the BAL fluid of young and aged mice infected with RSV (Fig. 6b). IL-6 levels increased significantly in the BAL fluid of aged mice from baseline to day 8 post-infection. At this time point, IL-6 levels were significantly higher in aged mice than in young mice. Levels of IL-6 decreased back to baseline again in aged mice by day 18. We took advantage of the natural variability in the response to infection in the aged mice group to understand the correlations between the muscle gene expression and airway IL-6. A significant, very strong, positive correlation was found between Atrogin (*Fbxo32*) and MuRF-1 (*Trim63*) expression (Fig. 6c). Strong, positive correlations were also found between Atrogin (*Fbxo32*) and MuRF-1 (*Trim63*) and IL-6 concentrations in the airways (Fig. 6d and e). We did not see significant associations with other measure of inflammation in the lung such as lung cell numbers.

## Discussion

In this study, we determined the impact of age on skeletal muscle in acute respiratory infection using a murine model of RSV infection. In this model, aged mice develop more severe disease following RSV infection than young mice, as reported by others [27–30]. The higher levels of RSV L-gene we detect by RT-PCR agrees with other reports of a higher viral load and delayed viral clearance in RSV infection of aged mice [27, 29, 38, 39].

We report that older mice infected with RSV showed significantly decreased skeletal muscle weight and muscle fiber size. Gene expression was altered in the skeletal muscle of aged mice in a pro-atrogenic pattern. The genes encoding the E3 ligases Atrogin-1 and MuRF-1 were upregulated following RSV infection in aged but not young mice. Myostatin can promote the gene expression of Atrogin-1 and MuRF-1, however, the decline in expression of the *Mstn* gene and lack of correlation with Atrogin-1 and MuRF-1 expression may suggest this is not the principal driver of loss of muscle mass in this model. Lower expression of *Mstn* in this model is, however, consistent with reductions in circulating myostatin seen in patients following surgery, in response to burn injury and with the inverse association of myostatin with CRP in chronic liver disease [40–43]. Pax7 is the marker for satellite cells and is required for muscle regeneration. Myogenin is expressed in myoblasts and suppresses Pax7, committing the cells to terminal differentiation. The lower *Pax7* expression at baseline in aged mice may reflect a reduced regenerative capacity due to fewer satellite cells. Sustained lower *Pax7* expression and higher *Myog* expression in aged mice may be the result of satellite cell activation and progression towards muscle fiber differentiation in the older mice following infection. However, gene expression of these factors may not fully reflect their activity and a detailed study of satellite cells in the elderly muscle following respiratory viral infection is needed.

Mechanisms leading to the aging-associated changes in skeletal muscle in our aged mouse model are likely to be multifactorial. Poor control of viral replication, leading to a higher viral load in the older mice, could drive more local inflammation in the lung, in combination with a more pro-inflammatory immune response with age. Elevated levels of inflammatory cytokines and biomarkers, including TNFα, IL-6 and CRP, have been associated with frailty in multiple studies and implicated as a potential mechanism in acute sarcopenia following respiratory infection [23, 24, 44]. We found a far greater production of IL-6 in the lungs of the aged mice during RSV infection and elevated IL-6 production has been reported in severe RSV infection in adults [45]. An enhanced local inflammatory response in the lungs may drive the weight loss and muscle loss in the mice, as supported by the correlation we find between lung IL-6 and changes to gene expression in the muscle in aged mice following infection. Our results are consistent with the high expression of IL-6 reported in aged mice infected with influenza and direct effects of IL-6 on atrogin expression in myotubes [46]. However, although weight and muscle loss may result from the direct actions of inflammatory mediators on the muscle, this correlation does not demonstrate a causal relationship. Other factors may contribute to the changes we observe in the muscle, for example local inflammation and lymphocyte infiltration into the muscle, as reported in influenza infection in aged mice [47] and we find elevated IL-6 gene expression in the muscle of aged but not young mice during RSV infection. We have not identified the cellular source of IL-6 in the lungs and muscle during RSV infection or the source of other mediators that promote muscle loss. We see increased protein synthesis as measured by puromycin incorporation, following infection, in young but not aged mice. This is consistent with lower levels of IGF-1, which can promote protein synthesis, in aged mice. Additionally, changes to activity or appetite, as seen in our model, which are also regulated by inflammatory cytokines, may contribute to weight and muscle loss. These results together suggest aging results in muscle wasting in elderly mice following RSV infection because of both higher muscle protein breakdown and lower protein synthesis. Furthermore, our data also demonstrate intrinsic differences in the muscle with age, for example, Atrogin-1 (*Fbxo32*) and *Pax7* gene expression were significantly different at baseline in the aged mice. Pre-existing differences in the muscles with age in turn may influence the response of the muscle to inflammatory stimuli.

The limitations of this work are primarily that this is a murine model and the direct applicability to human infection may be limited. All adults will have been infected with RSV throughout their lives and as such, unlike the aged mice used, will not be immunologically naïve. Older adults differ in the severity of RSV infection and the reasons why some adults develop severe RSV disease is not fully understood, and so may not be fully replicated in this model. Furthermore, most of our analyses was carried out at only two time points, d8 and d18 post infection, as well as in uninfected mice, and a more detailed time course could reveal changes occurring early in infection and during resolution of inflammation and muscle regeneration. In addition, only female mice were used and changes in muscle function were not measured. This model has the potential to reveal the key mechanisms that promote muscle wasting following RSV infection, for example, by blocking different inflammatory pathways, but such interventions were beyond the scope of this study.

This work may have wider implications for our understanding of the interaction of aging, infection, and frailty, which is of increasing clinical importance in an aging population. Muscle wasting is a natural component of aging. It is enhanced by inactivity and chronic disease and is associated with frailty and increased mortality. Therefore, the factors and mechanisms regulating muscle mass are of strong interest to a rapidly aging society. Age-related loss of muscle mass and strength (sarcopenia) is a central component of frailty. Frailty is itself associated with increased susceptibility to respiratory viral infection [1] and risk factors for severe RSV include frailty and chronic age associated diseases, including heart disease and COPD [48–53]. Existing sarcopenia is also associated with impaired immunity to influenza and worse outcomes in patients hospitalized with Covid-19 [54, 55]. There may be long term effects of respiratory infection on muscle and frailty. There is an increased long-term risk of all-cause mortality [56], and of cardiovascular events following hospitalization with RSV in adults [57] and following Covid-19, effects on the muscle can persist for years [10, 11]. In elderly people in particular, therefore, muscle wasting is associated with a downwards spiral of increased risk of falls and reduced activity, and further atrophy, frailty, and an increased risk of respiratory infection and all-cause mortality [58–61].

Our results demonstrate that aging alters muscle gene expression and protein synthesis, promoting muscle wasting in response to acute RSV infection. The data indicate that RSV infection of aged mice is a useful model for the study of the factors promoting enhanced muscle wasting in older age and for the development of inhibitors of such muscle loss. This model has the wider potential to yield insights into mechanisms of diseases where inflammation is a likely contributor to muscle loss and in older individuals, and will allow us to gain further mechanistic insights into the interaction between age, muscle, and respiratory infection, to develop treatments or prophylactics for infection-induced muscle wasting.

## Supplementary information

Below is the link to the electronic supplementary material.

Supplementary file1 (PDF 61360 KB)

## Data Availability

The data generated in this study are available from the corresponding author on reasonable request.
