# Supplementary material for: Aging enhances pro-atrogenic gene expression and skeletal muscle loss following respiratory syncytial virus infection
Source: GeroScience. 2024 Oct 2;47(2):1485–500. doi: 10.1007/s11357-024-01370-2 (PMC11978595; doi:10.1007/s11357-024-01370-2)
Supplement: Supplementary file 1 — Supplementary file1 (PDF 61360 KB) [file 11357_2024_1370_MOESM1_ESM.pdf]

**Supplementary Figures**

Young and aged female C57BL/6 mice were infected with RSV. Tibialis anterior muscles were isolated at d8 or d18 of infection and from uninfected mice. Figures show representative tiled images of a cross section of the tibialis anterior muscle. Colors indicate staining with antibodies to myosin heavy chain IIA (blue), myosin heavy chain IIB (green), myosin heavy chain I (yellow) and laminin (red). Scale bar = 500μm.

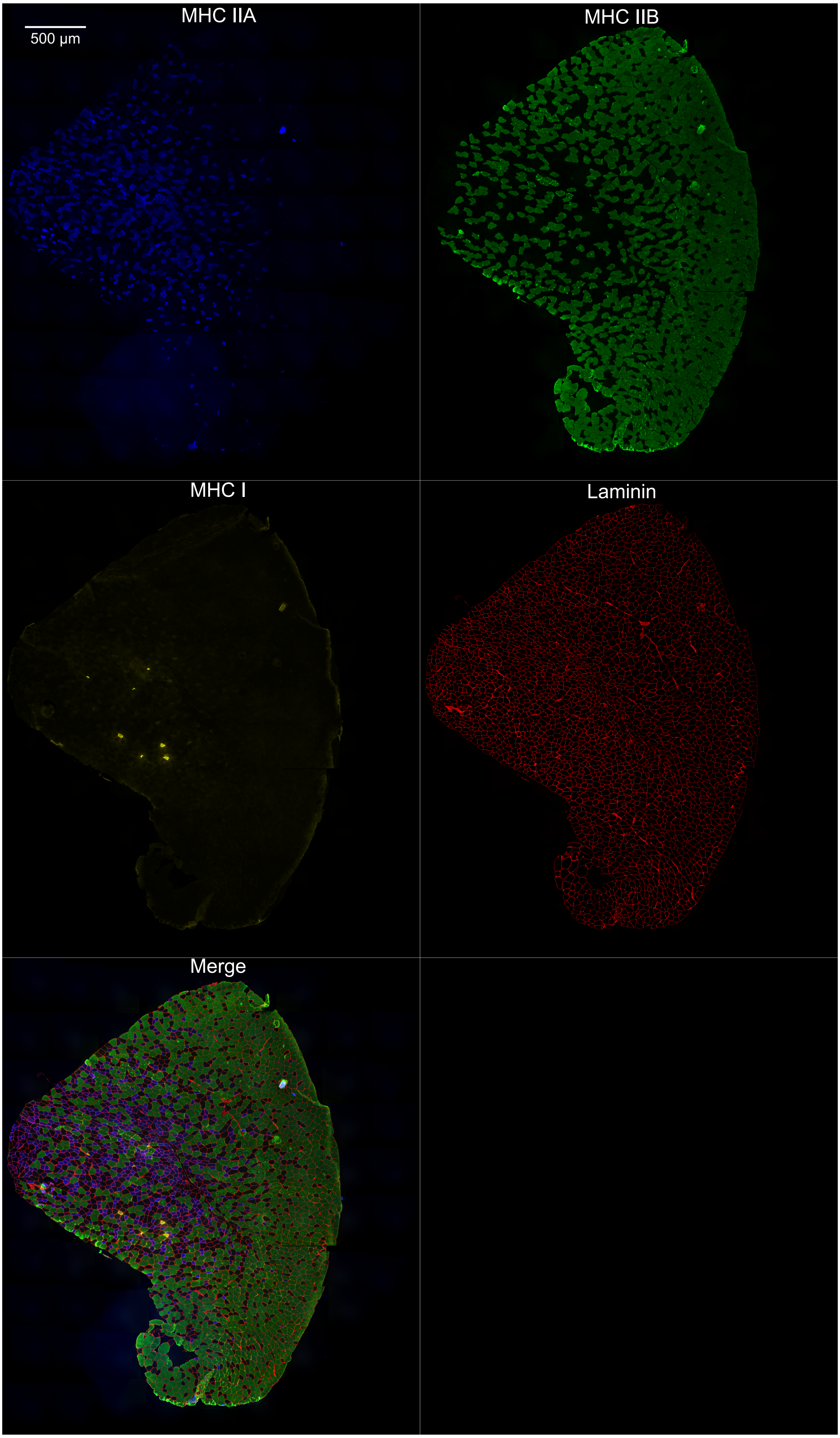


Young uninfected

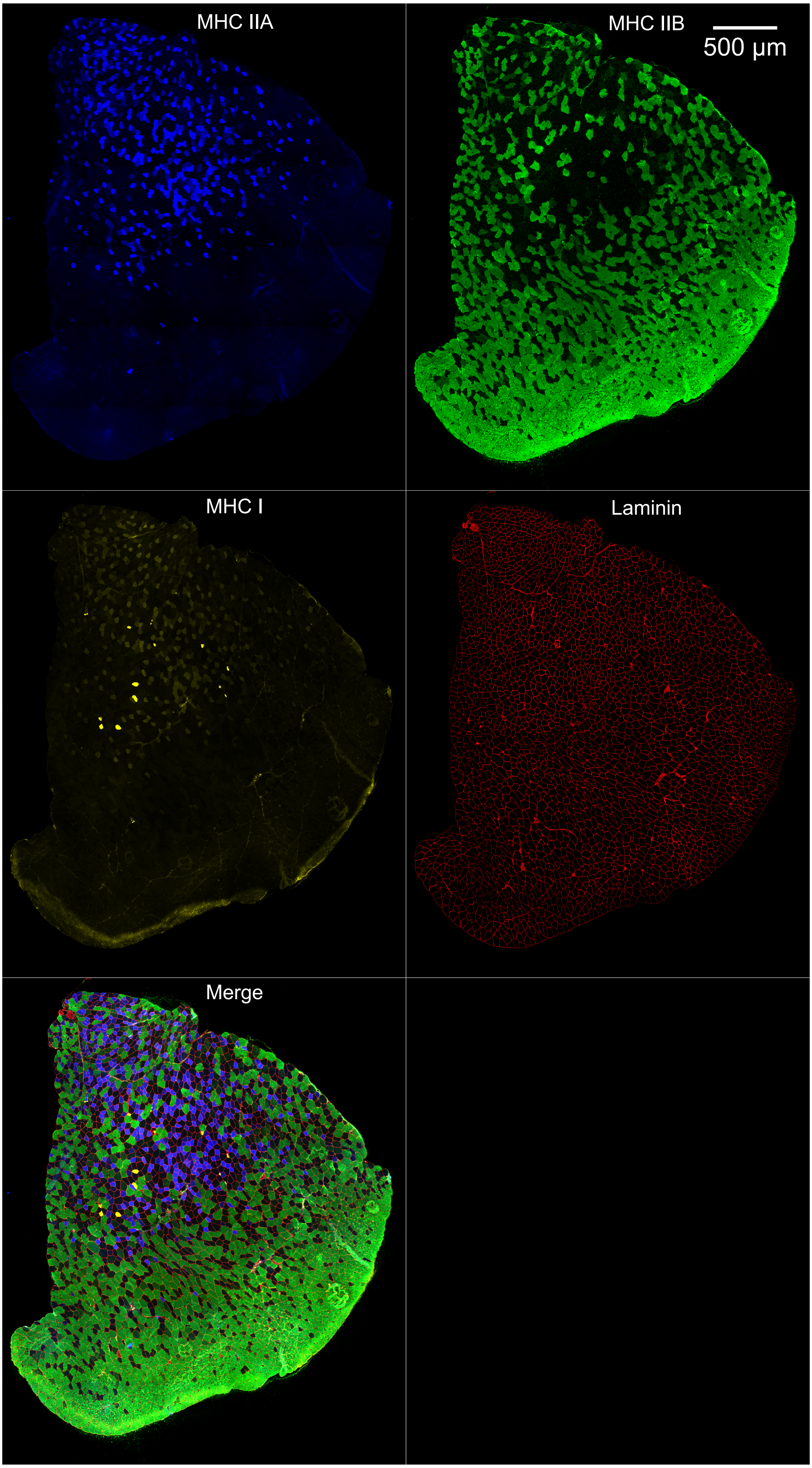


Aged uninfected

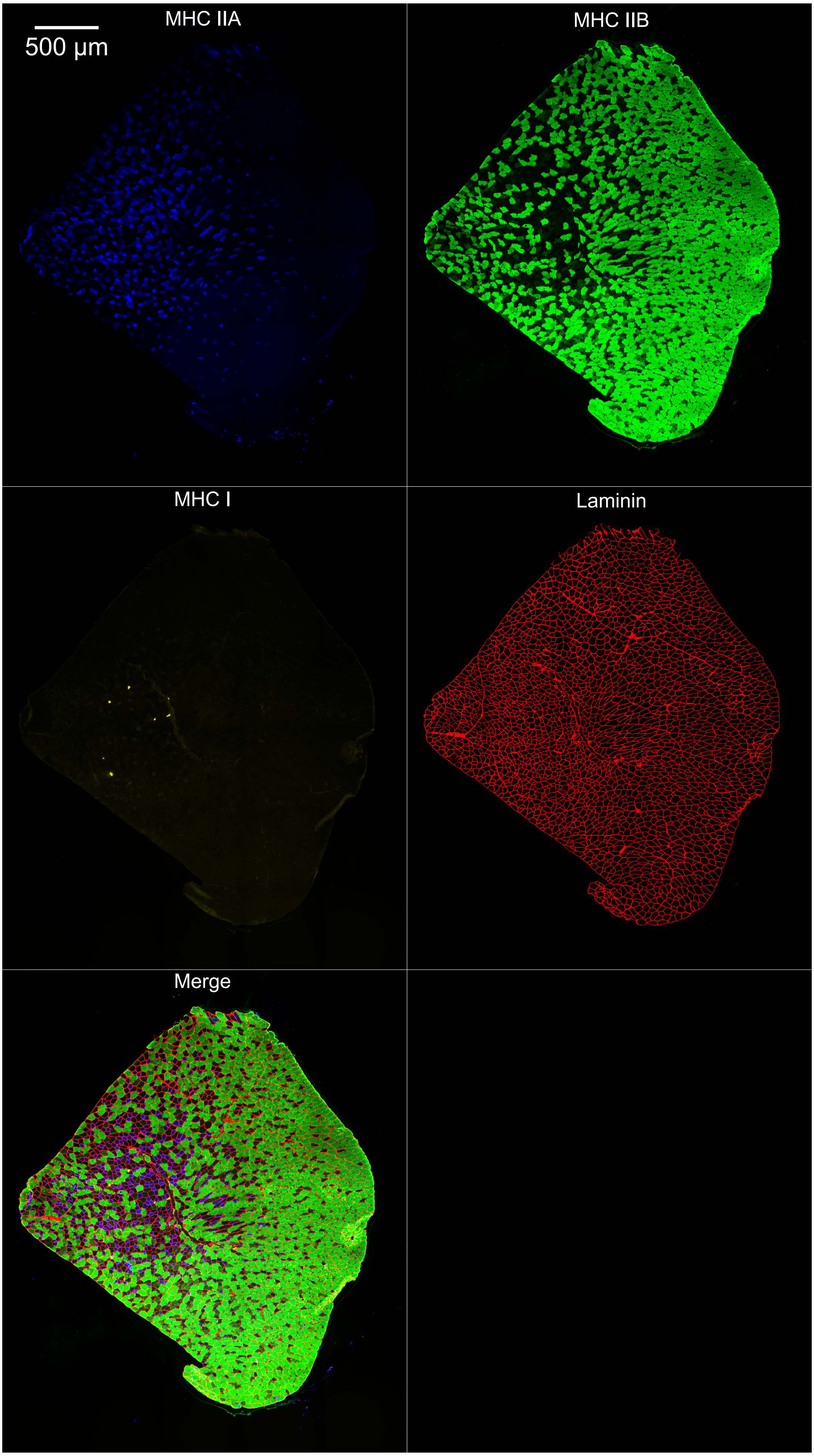


Young d8

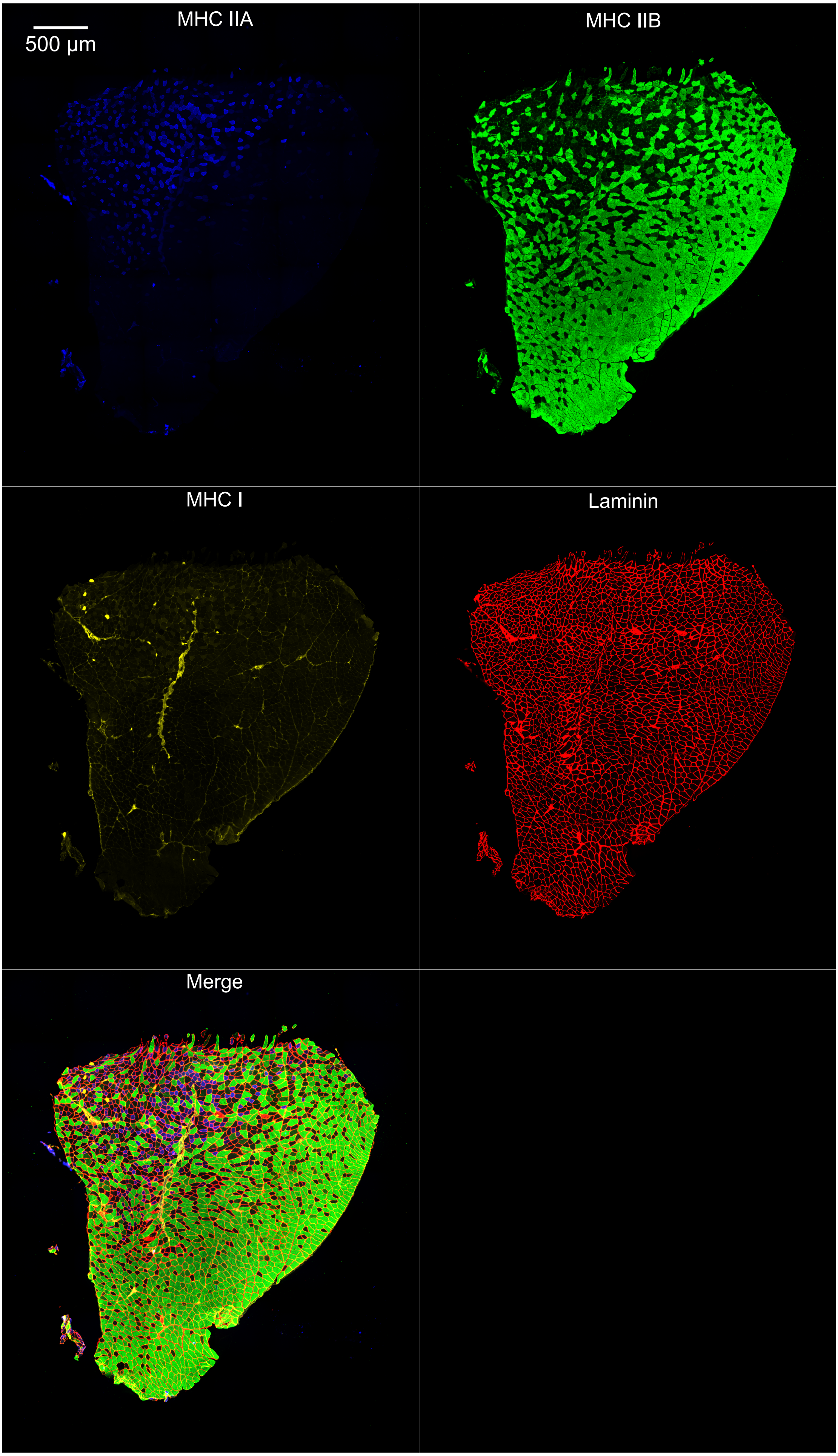


Aged d8

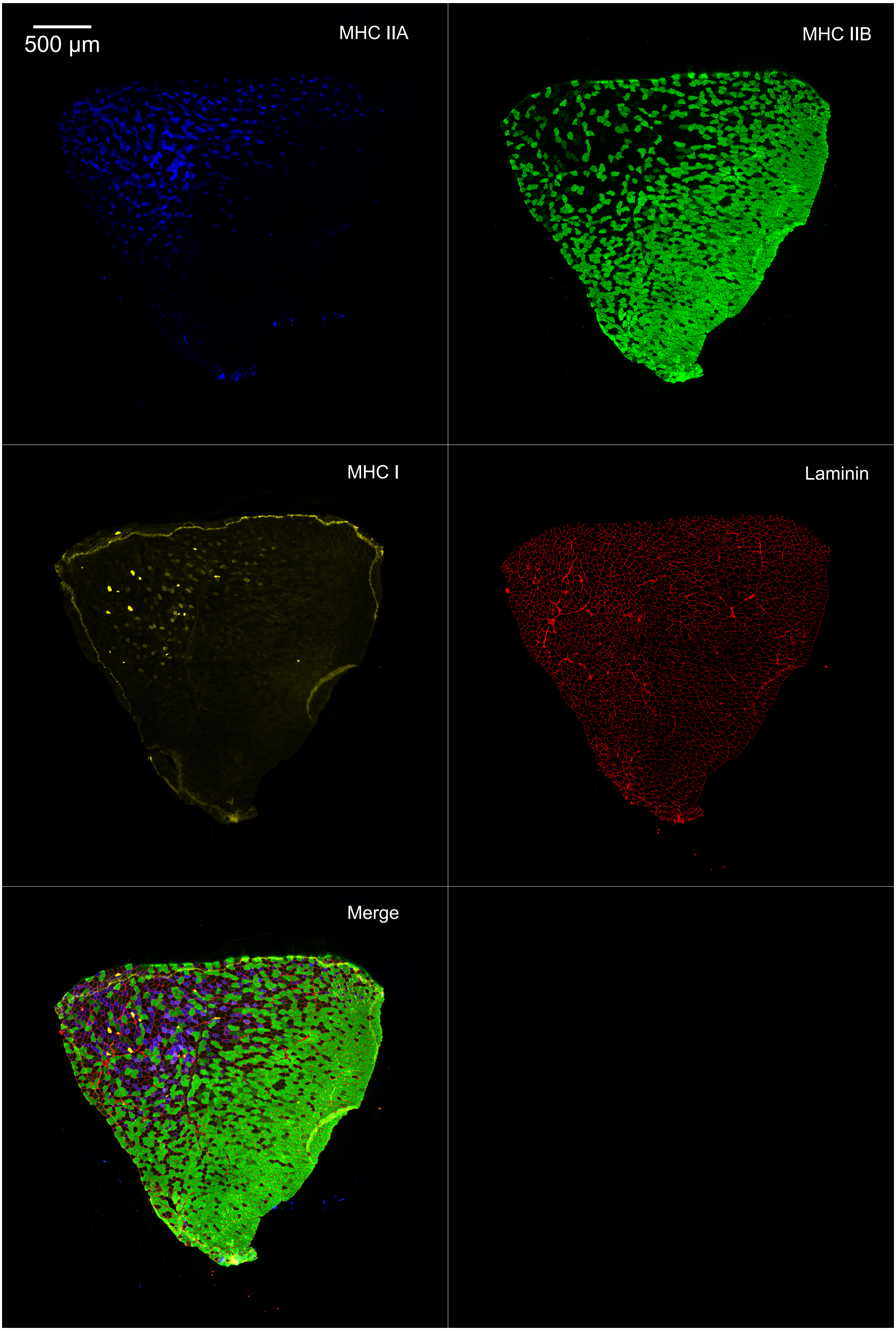


Young d18

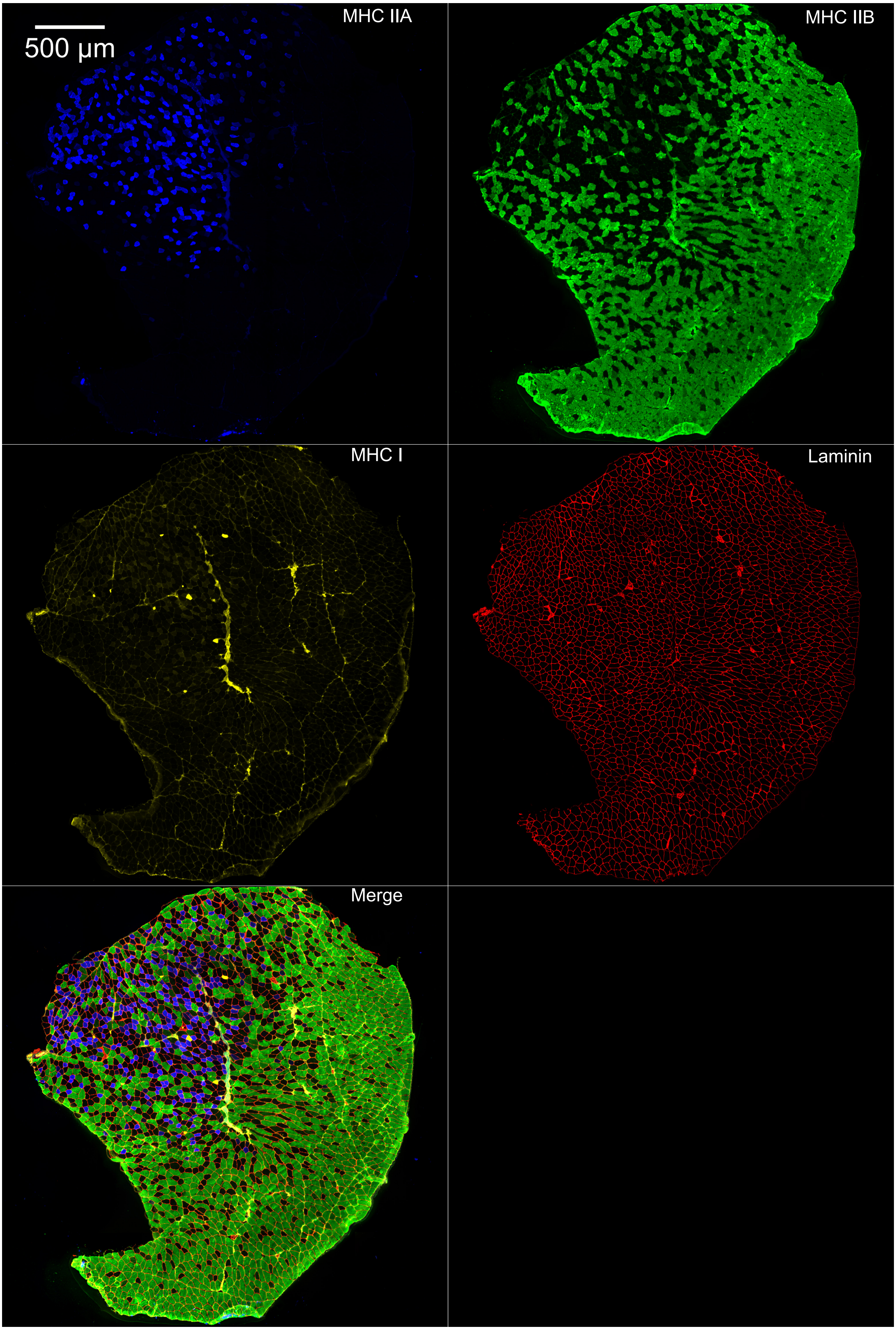


Aged d18
